# A Novel Anphevirus in *Aedes albopictus* Mosquitoes Is Distributed Worldwide and Interacts with the Host RNA Interference Pathway

**DOI:** 10.3390/v12111264

**Published:** 2020-11-06

**Authors:** Mosè Manni, Evgeny M. Zdobnov

**Affiliations:** 1 Department of Genetic Medicine and Development, University of Geneva Medical School, Rue Michel-Servet 1, 1206 Geneva, Switzerland; evgeny.zdobnov@unige.ch; 2 Swiss Institute of Bioinformatics, Rue Michel-Servet 1, 1206 Geneva, Switzerland

**Keywords:** virus discovery, mosquitoes, *Aedes albopictus*, *Anphevirus*, *Mononegavirales*, insect-specific viruses, small interfering RNA, PIWI-interacting RNA

## Abstract

The Asian tiger mosquito *Aedes albopictus* is a competent vector for several human arboviruses including dengue, chikungunya and Zika viruses. Mosquitoes also harbor insect-specific viruses (ISVs) that may modulate host physiology and potentially affect the transmission of viruses that are pathogenic to vertebrates, thus representing a potential tool for vector control strategies. In *Ae. albopictus* we identified a novel anphevirus (family *Xinmoviridae;* order *Mononegavirales*) provisionally designated here as Aedes albopictus anphevirus (AealbAV). AealbAV contains a ~12.4 kb genome that is highly divergent from currently known viruses but displays gene content and genomic organization typical of known anpheviruses. We identified AealbAV in several publicly available RNA-Seq datasets from different geographical regions both in laboratory colonies and field collected mosquitoes. Coding-complete genomes of AealbAV strains are highly similar worldwide (>96% nucleotide identity) and cluster according to the geographical origin of their hosts. AealbAV appears to be present in various body compartments and mosquito life stages, including eggs. We further detected AealbAV-derived vsiRNAs and vpiRNAs in publicly available miRNA-Seq libraries of *Ae. albopictus* and in samples experimentally coinfected with chikungunya virus. This suggests that AealbAV is targeted by the host RNA interference (RNAi) response, consistent with persistent virus replication. The discovery and characterization of AealbAV in *Ae. albopictus* will now allow us to identify its infection in mosquito populations and laboratory strains, and to assess its potential impact on *Ae. albopictus* physiology and ability to transmit arboviruses.

## 1. Introduction

The Asian tiger mosquito *Aedes albopictus* is increasing its spread worldwide and represents a serious threat to public health as it can transmit medically important arthropod-borne viruses (arboviruses) including dengue, chikungunya and Zika viruses [1,2,3]. Mosquitoes, like other insects, can also carry insect-specific viruses (ISVs) [4] which are not pathogenic to vertebrates. ISVs generally have a narrow host-range specificity and may be maintained in insect populations through vertical and/or horizontal transmission [5]. The presence of ISVs in mosquito populations or laboratory colonies may influence host physiology and arboviral infection, for example, via modulating the immune system. It has been shown that some ISVs in mosquitoes can affect replication and transmission of co-infecting pathogenic viruses [6,7,8,9] and be modulated by *Wolbachia* symbionts [10,11,12]. Therefore, ISVs might be potentially exploited in arbovirus transmission-blocking strategies [13]. The effect of ISV infections on mosquito physiology and symbionts may also be particularly relevant in the context of mosquito population control strategies such as the sterile insect technique (SIT) and the incompatible insect technique (IIT) that relies on the presence of the maternally inherited endosymbiotic bacterium *Wolbachia* [14]. Unrecognized ISV infections, on the other hand, could be a hidden variable that drives signal (for example, in expression data) when comparing ISV-infected and non-infected populations/strains or when evaluating vector competence and host-response to viral infections. Identifying ISVs, their specific hosts, and recognizing ISV infections is thus of primary importance in vector biology research.

We report the de novo assembly and annotation of a negative-sense RNA virus, provisionally named Aedes albopictus anphevirus (AealbAV), from the family *Xinmoviridae* (order *Mononegavirales*). The International Committee on Taxonomy of Viruses (ICTV) recently established the *Xinmoviridae* viral family to accommodate the genus *Anphevirus* [15]. All members of *Xinmoviridae* have monosegmented genomes of approximately 12 kb in length and encode 6/7 open reading frames (ORFs) that are sequentially transcribed by the RNA polymerase complex forming a characteristic transcription gradient [16]. We investigated AealbAV geographical and tissue distribution by assessing its presence in publicly available *Ae. albopictus* RNA-Seq datasets. We have further characterized the interaction of AealbAV with the *Ae. albopictus* immune system. Small RNAs, including small-interfering RNAs (siRNAs) and P-element-induced wimpy testis in *Drosophila* (PIWI)-interacting RNAs (piRNAs), regulate virus infection in mosquitoes [17,18]. We identified AealbAV-derived siRNAs and piRNA in miRNA-Seq libraries including in samples experimentally infected with chikungunya virus (CHIKV).

The discovery of AealbAV in *Ae. albopictus* will now permit its identification in populations and laboratory colonies of this important arboviral vector. Whether AealbAV has effects on other co-infecting viruses warrants further investigation, especially in the context of its potential use in control strategies.

## 2. Materials and Methods

### 2.1. Virus Discovery

In total, 523 RNA-Seq and microRNA-Seq libraries of *Ae. albopictus* were downloaded from the NCBI Short Read Archive (SRA) (as of December 2019) and converted to FASTQ files using the NCBI SRA toolkit (https://www.ncbi.nlm.nih.gov/sra/docs/toolkitsoft/) [19]. Mosquito sequences were removed by filtering reads matching *Ae. albopictus* CDSs (from assembly GCA_001444175.2) [20] and genome assembly (GCA_001876365.2) [21] with bbduk from the BBTools suite v38.71 (sourceforge.net/projects/bbmap/) using a *k-*mer size of 31 nucleotides. The same program was used for removing reads matching common artifacts and homopolymers, and to trim Illumina adapter sequences. For the de novo assembly of libraries, low quality reads containing bases with a Phred quality score below 16 or reads with an average quality below 20 were removed. Remaining reads of each individual library (which might correspond to sequences from host-associated organisms) were de novo assembled using SPAdes genome assembler version v3.11.1 [22]. Resulting contigs from all libraries were clustered with CD-HIT v4.8.1 [23]. AealbAV sequences were discovered when a pattern emerged from clustering contigs: highly similar contigs (>96% nucleotide identity) with high coverage were assembled from several libraries. We further investigated these sequences and compared them to the non-redundant protein (nr, May 2020 release) and nucleotide (nt, May 2020 release) NCBI databases using Diamond v0.9.29 (e-value of 1 × 10^−4^) [24] and BLASTn of the BLAST+ suite [25], respectively.

AealbAV genome sequences were validated by mapping reads back to the assembled contigs using bbmap (sourceforge.net/projects/bbmap/) (minid = 0.94, maxindel = 6), which was also used for calculating various mapping statistics such as the depth and breadth of coverage. The SAM files generated from the mapping were converted into ordered BAM files using Samtools v1.9 [26]. AealbAV assemblies were manually checked and visualized using the Integrative Genomics Viewer (IGV) v2.8.0 [27]. The Gviz package [28] in R studio (http://www.rstudio.com/) was used to create the coverage plots of each AealbAV assembly using the corresponding BAM files. All AealbAV assemblies across samples are >96% identical at nucleotide level along the full-length sequence thus representing the same viral species. For convenience, one genome was chosen as representative to carry out annotation, motif discovery, phylogeny and miRNA profiling. AealbAV genomes were also assembled from miRNA-Seq libraries with Virus Detect v1.7 [29] using AealbAV representative genome as reference. Reads from libraries generating AealbAV assemblies were also reassembled without subtracting mosquito reads to further check the consistency of the AealbAV sequences. Mafft [30] was used to compute alignments of coding-complete AealbAV genomes. Where it was not possible to assemble a coding-complete genome, we considered a library as AealbAV-positive if the mapping reads covered >50% of the AealbAV genome. Table S1 shows the assembly and/or mapping statistics of each data set.

### 2.2. Virus Genome Annotation

ORFs were predicted using the NCBI Open Reading Frame Finder (https://www.ncbi.nlm.nih.gov/orffinder/) with a minimum ORF length of 75 nucleotides using the standard genetic code. Predicted protein sequences were compared to the NCBI non-redundant protein database (nr) using BLASTP [25] and analyzed for protein domains using the NCBI Conserved Domain Search Service [31], HMMER (https://toolkit.tuebingen.mpg.de/tools/hmmer) and HHPred tool (https://toolkit.tuebingen.mpg.de/#/tools/hhpred). Putative transmembrane domains were identified using TMHMM web server (https://services.healthtech.dtu.dk/service.php?TMHMM-2.0) [32] and TOPCONS web server (http://topcons.cbr.su.se/) [33]. Glycosylation sites were predicted by the NetNGlyc 1.0/NetOGlyc 4.0 server (https://services.healthtech.dtu.dk/). Sequence motifs were searched using the MEME Suite v5.1.1 [34].

### 2.3. Phylogenetic Analysis 

Phylogenetic analysis was performed using the RNA-dependent RNA polymerase (RdRp) proteins of AealbAV and representatives of the *Xinmoviridae* and *Nyamiviridae* viral families. The sequences were aligned using MAFFT v7.450 (https://mat.cbrc.jp/alignment/server/) [30] employing the G-INS-i algorithm, and trimmed with trimAL v3.4.1 [35] with option “-automated1”. The best-fit amino acid substitution model was estimated with ProtTest v3.4.2 [36] (-S 2 -all-distributions). Maximum likelihood phylogenetic tree was computed with IQ-TREE v1.6.10 [37] with 1000 bootstrap resampling. Phylogenetic tree was drawn with FigTree v1.4.4 (http://tree.bio.ed.ac.uk/software/figtree/) and annotated in Inkscape (https://inkscape.org/).

### 2.4. Virus-Derived Small RNA Profile 

Analysis of small-RNA reads was performed by mapping adapter-trimmed reads to the AealbAV reference genome using bbmap (sourceforge.net/projects/bbmap/). Length distributions and mapping profiles of vsiRNAs (21 nt) and vpiRNAs (24–29 nt) were computed using bbmap and visualized in R studio with the ggplot2 package. The program PingPongPro v1 [38] was used to examine the preferred overlap between vpiRNAs reads mapping on opposite strands of AealbAV. The analysis of the frequency of nucleotide usage in 27-nt vpiRNAs was performed using WebLogo (http://weblogo.threeplusone.com/) [39]. 

## 3. Results 

We identified and characterized the genomic sequence of a novel negative-strand RNA virus infecting *Ae. albopictus* samples worldwide. We assessed its geographical and tissue distributions by analyzing publicly available *Ae. albopictus* RNA-Seq and miRNA-Seq datasets. As detailed below, gene content, genome organization and phylogenetic placement support the classification of this virus as belonging to the genus *Anphevirus* from the *Xinmoviridae* family (order *Mononegavirales*). Based on the shared features with known anpheviruses, we provisionally named this virus Aedes albopictus anphevirus (AealbAV).

### 3.1. Identification and Assembly of AealbAV Genome 

We downloaded the 523 *Ae. albopictus* RNA-Seq and miRNA-Seq datasets publicly available from the Sequence Read Archive (SRA) deposited until December 2019. After removing sequences of *Ae. albopictus* and low quality reads, the remaining reads of each paired-end RNA-Seq library were de novo assembled into contigs. The resulting contigs from all individual libraries were clustered at >90% nucleotide identity. AealbAV genome was discovered by looking at the distribution of highly similar contigs with high coverage across several libraries. Specifically, contigs from 72 libraries clustered together at >96% nucleotide identity and displayed high coverage (>100×) in several libraries (Table S1). Portions of these contigs shared ~40% identity at amino acid level with the RdRp protein of known anpheviruses (nr, May 2020 release).

From 23 of the 72 libraries, viral coding-complete AealbAV genomes were recovered in a single contig of around 12 kb, a typical size observed in known anpheviruses. AealbAV assemblies were validated through remapping to the draft genomes and were manually inspected. The average depth of coverage of AealbAV varied between 17 × (SRR652120) to 332 × (SRR458470) (Table 1, Table S1) in libraries generating coding-complete genomes. AealbAV genomes were assembled into partial assemblies in the remaining 47 paired-end libraries due to lower read coverage (Table S1). The high AealbAV depth and breadth of coverage in some miRNA-Seq libraries (depth of coverage: > 100 ×; breadth of coverage: 100%) allowed us to also assemble coding-complete genomes from small RNA data (Table 1, Table S1).

Additionally, we took advantage of recently published sequencing libraries to expand the assessment of AealbAV in wild-caught mosquitoes collected in Italy, Greece and Thailand (BioProject: PRJNA602498, 2020) [40] as well as in Switzerland (SRA accession: SRP266553, 2020) [41]. We assembled full AealbAV genomes from these libraries except for the Greek sample in which AealbAV appears to be absent (Table 1, Table S1). 

The AealbAV coding-complete genomes assembled in this study shared >96% nucleotide identity over the full-length sequence and thus we consider these genomes as deriving from the same viral species. For convenience, 1 representative AealbAV genome (12,436 pb in size; 312× depth of coverage; assembled from library SRR458463) was chosen to carry out subsequent analyses. The representative AealbAV genome has been deposited in GenBank under the accession number MW147277. Predicted proteins were assigned to identifiers QOW17623-QOW17629. The other AealbAV genome sequences de novo assembled from the SRA data sets can be found at https://github.com/bio-mmanni/AealbAV_genomes.

Nucleotide sequence homology to other viruses were not detected in the NCBI database (nt, May 2020 release) when using BLASTn megablast, whereas a small portion of approximately 800 nucleotides (8% query coverage) gave hits to sequences of known anpheviruses at 74% nucleotide identity using BLASTn. During preparation of this manuscript the sequence of a novel virus named Serbia mononega-like virus 1 identified in *Culex pipiens* was published [42]. This virus shares approximately 70% amino acid identity with AealbAV in two genomic regions corresponding to a putative glycoprotein and the RdRp protein (Table 2). 

We queried the de novo assembled AealbAV genome against the transcriptome sequence archive (TSA) and expressed sequenced tags (ESTs) deposited at NCBI using BLASTn and tBLASTx to check whether AealbAV sequences were present in assembled transcriptomes or EST libraries. We found AealbAV hits small contigs (<16% query coverage) of the *Ae. albopictus* oocyte transcriptome from a laboratory colony of the Georgetown University [43], and of the *Ae. albopictus* antennal transcriptome of an Italian strain [44]. We further queried transcriptomes that are not deposited in NCBI. We found unannotated contigs matching AealbAV in a transcriptome from the Georgetown University [45] and an antennal transcriptome of wild mosquito populations [40], indicating AealbAV sequences went unnoticed in these transcriptomes likely due to its high divergence to known viral sequences.

### 3.2. Genomic Organization, Gene Content and RNA Motifs of AealbAV

**Figure 1:**
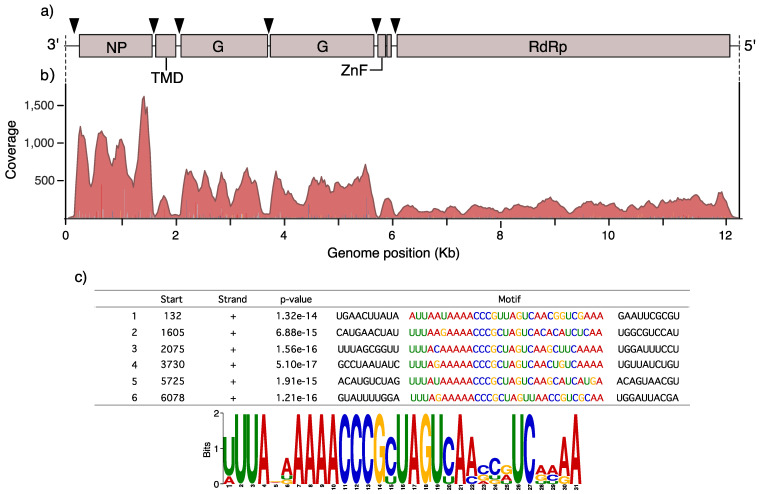
Aedes albopictus anphevirus (AealbAV) genome organization and conserved motif. (**a**) Genomic organization of AealbAV. Predicted ORFs are indicated with boxes. Positions of the 31-nt conserved motif are indicated by triangles. NP, nucleoprotein; TMD, Transmembrane domain; G, glycoproteins; ZnF, zinc-like finger; RdRP, RNA-dependent RNA polymerase. (**b**) Example of a mapping profile from 1 library (SRR458463) displaying the characteristic 3′–5′ transcription gradient typical of replicating monenegaviruses. (**c**) Sequences and logo of the 31-nt conserved motif as estimated by MEME.

AealbAV encodes for 7 putative Open Reading Frames (ORFs) including a putative nucleoprotein (N), a small transmembrane protein (STM), two putative glycoproteins (G1 and G2), a small ZnF protein (ZnF) and a large RdRp protein (L). These are positioned in the typical order found in other anpheviruses: 3′-N-STM-G1-G2-ZnF-L-5′ (Figure 1a). An additional small putative ORF is present between the ZnF and L proteins as observed in some anpheviruses [46]. More details on each ORF can be found in the supplementary note. Coverage analysis of AealbAV shows a transcription gradient with a reduction in transcriptional activity in the direction of 3′–5′ with ORF7 as the least transcribed gene (Figure 1b), following a pattern seen in other replicating mononegaviruses including Aedes anphevirus [46]. 

Using MEME to search for overrepresented 6- to 50-nt motifs we found a 31-nt motif (3′-UUUANAAAAACCCGCUAGUCAASCRUCRMAA-5′) repeated 6 times on the AealbAV genome (Figure 1a,c). These motifs are located in intergenic regions in proximity to the predicted ORFs suggesting they might function as promoters.

### 3.3. AealbAV Is Highly Divergent from Currently Known Anpheviruses

**Figure 2:**
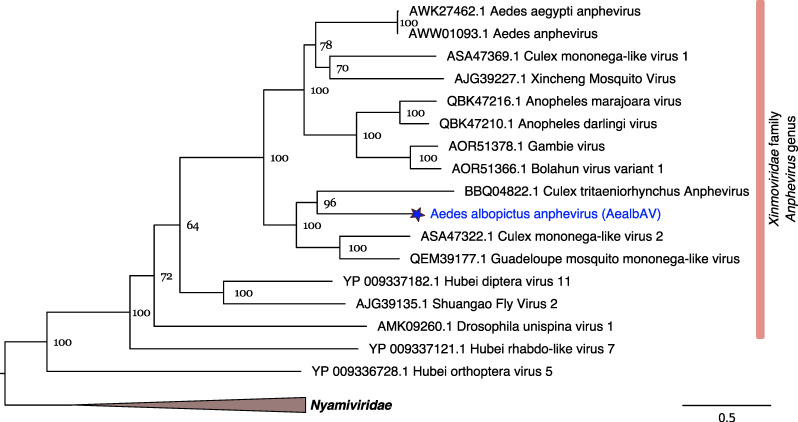
RdRp phylogeny of AealbAV and members of the *Xinmoviridae* and *Nyamiviridae* (Figure 1. bootstraps were performed). Branch lengths represent expected numbers of substitutions per amino acid site.

AealbAV genome is highly divergent from known anphevirus genomes. In the genomic region of the RdRp gene, AealbAV shares 69% amino acid identity with the recently discovered Serbia mononega-like virus 1, and between 37 and 42% identity with other anpheviruses (Table 2). A phylogenetic analysis using RdRp proteins of mononegaviruses places AealbAV within the recently established family of *Xinmoviridae* (Figure 2). The International Committee on Taxonomy of Viruses (ICTV) recently established this viral family to accommodate viruses of the floating genus *Anphevirus*, that is currently the sole genus of this family [15]. According to the last ICTV report, there are 8 officially classified viral species within *Xinmoviridae* [47]. Three of them were identified in mosquito species: the Xincheng mosquito virus (XcMV) from *A. sinensis* [48], Aedes anphevirus from *Ae. aegypti* [46], and Bolahun virus variant 2 from West African *Anopheles gambiae* mosquitoes [49]. Anphevirus-like genomes have also been described from other mosquito species including Australian *Culex* mosquitoes [50], Amazonian anophelines *A. marajoara* and *A. darlingi* [51], Japanese *Culex tritaeniorhynchus* [52], and from Serbian *Culex pipiens* [42]. AealbAV is more closely related to viruses described in *Culex* mosquitoes (Culex tritaeniorhynchus anphevirus, and Culex mononega-like virus 2) then to Aedes anphevirus isolated from *Ae. aegypti*, which in turn, is more closely related to Culex mononega-like virus 1 (Figure 2). Given the rate of divergence observed among currently known anpheviruses, it is likely that this viral clade contains many viral species yet to be described.

### 3.4. AealbAV Is Distributed Worldwide in Ae. albopictus Laboratory Colonies and Wild Populations

**Figure 3:**
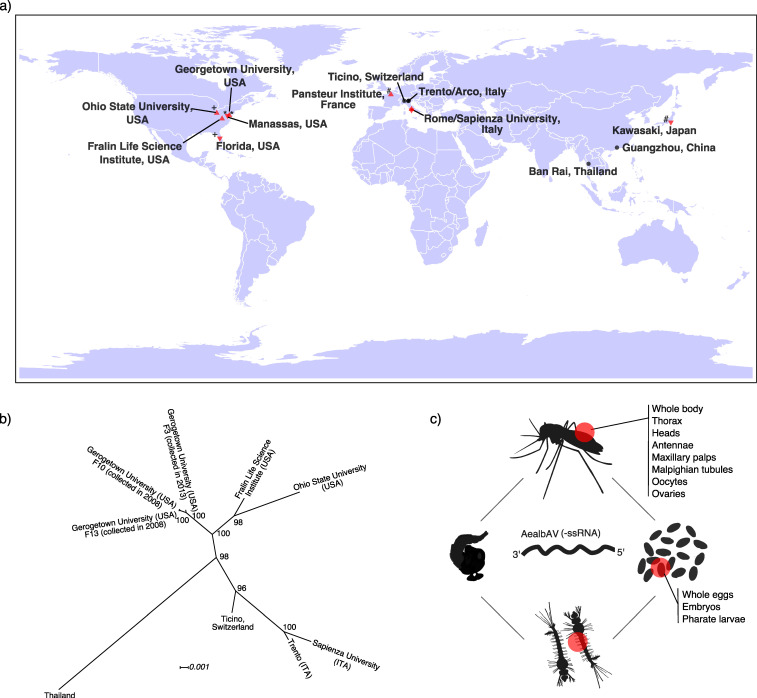
Geographical and tissue distribution of AealbAV and phylogenetic relationship of AealbAV strains. (**a**) AealbAV is present worldwide in *Ae. albopictus* laboratory colonies and wild-caught mosquitoes. Locations of mosquito collection from RNA-Seq and miRNA-Seq libraries that harbor AealbAV are shown. Red up-pointing triangles refer to the location of the laboratory colonies; Red down-pointing triangles refer to the collection sites of the samples used to establish the laboratory colonies (if this information was available). An identical symbol nearby the triangles specifies the link between collection and colony sites (also see Table 1); Black points refer to the collection sites of wild-caught mosquitoes. (**b**) Phylogeny of representatives AealbAV genomes from different geographical locations. AealbAV genomes cluster by the geographical origin of their mosquito hosts. AealbAV strain from Thailand is the most differentiated. The unrooted Maximum likelihood phylogeny was constructed from alignments of nucleotide sequences of AealbAV strains using a GTR + F + I model with 1000 bootstraps. Node values correspond to bootstrap support. Branch lengths represent expected numbers of substitutions per nucleotide site. (**c**) AealbAV sequences were found in *Ae. albopictus* libraries derived from various life stages including embryos/eggs, and tissues, including oocytes and ovaries.

We identified AealbAV reads in RNA-Seq and miRNA-Seq libraries of *Ae. albopictus* from different geographical locations from both laboratory strain and field-collected mosquitoes (Table 1, Figure 3a). AealbAV was present in libraries of *Ae. albopictus* laboratory colonies from: (i) the Georgetown University (USA, 55 libraries [SRP012105, SRP018112, SRP050258, SRP096579, SRP007714]) established from larvae and pupae collected in Manassas (USA) in 2008, 2010 and 2013 [43,45,53,54,55]; (ii) the Fralin Life Science Institute (USA, 5 libraries [SRP008316]) [56]; (iii) the Sapienza University (Italy, 8 samples [SRP071220]) established from eggs collected in Rome (Italy) in 2012 [44]; (iv) the Pasteur Institute (France, 2 samples [SRP228299]) established from mosquitoes collected in Kawasaki (Japan) in 2008 [57]; and (v) the Ohio State University (USA, 38 libraries [SRP056407 SRP034701]) from the *Ae. albopictus* MRA-804 strain which derives from samples collected in Florida (USA) [58,59] (Table 1, Figure 3a). It is likely that the wild source populations of these laboratory strains were infected with AealbAV and that the virus was maintained in these colonies likely through vertical transmission. This is also corroborated by the samples from the Georgetown University where AealbAV was found in libraries from different generations (F5, F10, F13) of the same colony established from samples collected in Manassas in 2008 [43,45,55], and from two different colonies (F12, F3) also established from samples collected in Manassas but in 2010 and 2013, respectively [53,54] (Table 1). This suggests that AealbAV is also likely maintained in the wild source population of Manassas. We also identified AealbAV in wild-caught *Ae. albopictus* mosquitoes collected in Southern Switzerland (14 libraries [SRP266553]) [41] as well as in China (15 libraries [SRP188743]), Italy and Thailand (3 library [PRJNA602498]) [40] (Table 1, Figure 3a). It is worth noting that AealbAV was absent in wild-caught mosquitoes collected in Athens, Greece (1 library [PRJNA602498]).

To assess if AealbAV could infect *Ae. aegypti* we additionally searched for AealbAV in all the 829 publicly available RNA-Seq libraries of *Ae. aegypti* (as of December 2019). We did not find evidence for its presence in these data sets.

### 3.5. Ae. albopictus Mosquitoes Worldwide Carry Highly Similar AealbAV Strains

Coding-complete genomes of AealbAV assembled from laboratory colonies from the USA and Italy, and wild populations from Italy, Switzerland and Thailand show high similarity with an average pairwise nucleotide identity among all strains of 98.2%. The two more divergent genomes are from Italy (Rome) and Thailand with 96.7% average nucleotide identity. A phylogenetic tree constructed from representative genomic sequences shows that the strains cluster by the geographic origin of the mosquito hosts (Figure 3b). AealbAV from the USA cluster together whereas the Swiss and Italian strains are more closely related. The sample originated from Thailand is more divergent and might reflect the ancestral origin of Thai mosquitoes. These relationships seem to reflect the recent colonization history of *Ae. albopictus* which has spread in recent decades from native areas (e.g., Thailand) to newly colonized areas, including North America and Europe [60,61].

### 3.6. Eggs and Various Tissues of Ae. albopictus Harbor AealbAV

AealbAV reads/contigs were identified in samples from different mosquito life stages, namely in whole eggs/embryos, larvae and adults (both males and females). The libraries from embryos of 72–78 h and 135–141 h had on average the highest depth of coverage (max coverage: 361.5×) (Table S1). Libraries from various body compartments contain AealbAV, including whole bodies (male/female), heads, thorax, antennae, maxillary palps, female Malpighian tubules, oocytes and ovaries (Table 1, Table S1, Figure 3c). The presence of AealbAV in embryos, oocytes and ovaries further suggests that AealbAV is vertically transmitted. In our analysis we did not find evidence for AealbAV presence in RNA-Seq libraries of *Ae. albopictus* cell lines.

### 3.7. Ae. albopictus RNAi Response Targets AealbAV

**Figure 4:**
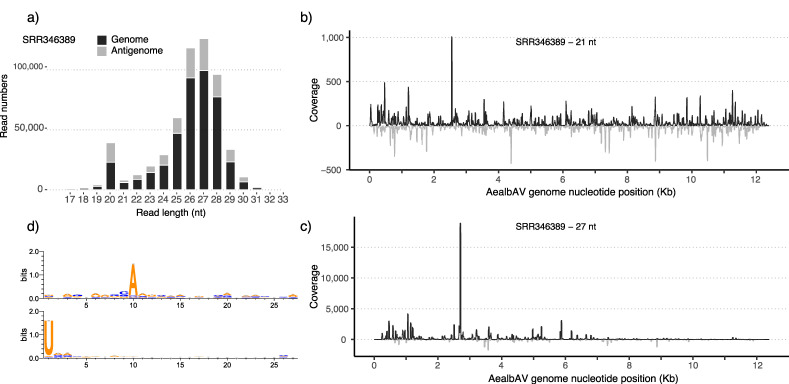
AealbAV-specific miRNAs in *Ae. albopictus* samples infected with CHIKV. (**a**) Size distribution of miRNAs mapping to the AealbAV genome (black) and antigenome (grey). (**b**) Mapping profiles of the 21-nt reads representing AealbAV-derived siRNAs and (**c**) the 27-nt reads representing AealbAV-derived piRNAs. (**d**) Relative nucleotide frequency and conservation of the 27-nt vpiRNAs that mapped to the genome (upper panel) and the antigenome (bottom panel) of AealbAV. AealbAV-derived vpiRNAs display the characteristic piRNA ping-pong signature with Adenine at position 10 for the genomic strand and Uridine at position 1 for the antigenomic strand.

Interestingly, we identified reads from miRNA sequencing datasets mapping to AealbAV genome (Table 1, Table S1). AealbAV miRNA reads were identified in samples of *Ae. albopictus* experimentally infected with CHIKV (SRR346385-SRR346389) [56]. Mapping miRNA reads covered 90 to 100% of AealbAV genome in these samples (Table S1). We also identified AealbAV miRNAs in *Ae. albopictus* pharate larvae (embryonated eggs) libraries from the Georgetown University (SRR5168326-SRR5168333) [54] (coverage: 1.1 to 2.5×; breadth of coverage: 52.7 to 74%). It is important to notice that we identified AealbAV sequences from both miRNA and RNA-seq data generated from this colony. We also found high levels (depth of coverage: 49.8×; breadth of coverage: 99%) of miRNAs mapping to AealbAV in a recently published dataset (SRR11213090) of *Ae. albopictus* experimentally infected with three distinct ISVs, namely, Aedes flavivirus, Menghai rhabdovirus and Shinobi tetravirus [57]. Overall, these data suggested that AealbAV may interact with the *Ae. albopictus* RNAi pathway. Therefore, we further investigated the possible mosquito RNAi response against AealbAV by analyzing the composition of the miRNA reads mapping to AealbAV using the published datasets of *Ae. albopictus* experimentally infected with CHIKV (SRR346385-SRR346389) [56]. For each library, adapter-trimmed reads were mapped against the AealbAV genome. AealbAV-derived miRNA reads ranged from 0.34% (library SRR346385) to 2.0% (library SRR346389) of the total sequenced clean reads. Figure 4 reports reads for library SRR346389 mapping to both the genome and the anti-genome of AealbAV (Figure 4a). Mapping profiles for the other libraries are reported in Figure S1. Mapping reads showed a length distribution with typical peaks at 21 nt and 27/28 nt likely corresponding to viral small interfering RNAs (vsiRNAs) and vpiRNAs respectively. Mapping reads of 21 and 27 nt were extracted and re-mapped to the genome. The 21-nt fraction of vsiRNAs targeted both the genome and anti-genome of AealbAV with a slight bias toward the genomic strand (Figure 4b). The 21-nt peak and the coverage along all AealbAV genome on both senses indicates that these AealbAV-derived siRNAs are likely produced by Dicer−2-mediated cleavage of the dsRNAs produced during virus replication.

The 24-nt to 30-nt fraction of small RNAs mapping to AealbAV displayed a size distribution characteristic of piRNAs with a peak at 27/28-nt. Contrary to 21-nt viRNA, these 27-nt AealbAV-derived piRNAs mostly map to the genomic sequence with few hot spots (Figure 4c). The 27 nt vpiRNAs display a clear piRNA ping-pong signature with a A_10_ bias in reads mapping to the genomic strand and a U_1_ bias in reads mapping to the antigenome (Figure 4d). We also observed the 10-nt overlap signature between sense and antisense vpiRNAs as expected for piRNAs produced by the ping-pong amplification cycle. 

### 3.8. Putative Anphevirus-Like NIRVs in Ae. albopictus Genomes

Several non-retroviral integrated RNA virus sequences (NIRVs) have been described in mosquito genomes including *Ae. albopictus* [62,63,64]. We found putative anphevirus-like insertions in *Ae. albopictus* genomes. There were several hits to AealbAV ranging from 67 to 76% nucleotide identity spanning regions between 100 and 2604 nt in three *Ae. albopictus* genome assemblies (Table S2). Putative insertions in *Ae. albopictus* Rimini strain were shorter and hit several contigs likely due to the high fragmented state of this assembly. Twenty-one putative insertions were found in the latest *Ae. albopictus* assembly (GCA_001444175.2) on nine scaffolds (Table S2). For example, scaffold NW_021838798.1 harbor the first anphevirus-like insertions (1827 bp) at approximately 16 Mb, a second insertion (2604 bp) around 48.4 Mb, and 6 anphevirus-like insertions (1117, 420, 440, 692, 614 and 297 bp) around 74.5 Mb. Further work would be required to understand whether these sequences are bona fide insertions. 

## 4. Discussion

We identified and characterized a negative-sense RNA virus that infects *Ae. albopictus* laboratory colonies and wild populations worldwide. We provisionally named this virus Aedes albopictus anphevirus (AealbAV) on the basis of the phylogenetic evidence, gene content and genomic organization that are typical of known anpheviruses. We de novo assembled or mapped AealbAV sequences from several publicly available *Ae. albopictus* RNA-Seq and miRNA-Seq libraries. All coding-complete genomes of strains from different geographical locations display intact ORFs and share high pairwise nucleotide identity (>96%) along the full genomic sequence. This little variation among strains may reflect the recent and rapid colonization history of *Ae. albopictus* in the past few decades, implying that AealbAV strains have not diverged substantially over this relatively short time period. Nonetheless, phylogenetic analysis cluster the strains by the geographic origin of the mosquito hosts, with AealbAV from Thailand being more divergent from the others, likely reflecting the ancestral origin of Thai mosquitoes. Future studies assessing the presence of AealbAV in native and newly established populations should provide more insights into the co-evolutionary history of this anphevirus with *Ae. albopictus*. 

The conserved 31-nt RNA motif localized in proximity of 6 ORFs likely functions as a promoter. Similar conserved motifs were also described in Aedes anphevirus [46] and anpheviruses-like species identified in Amazonian anophelines *A. marajoara* and *A. darlingi* [51], suggesting it is a conserved feature of this viral clade. The coverage pattern of AealbAV shows the typical 3′–5′ transcription gradient, characteristic of infecting mononegaviruses. This is explained by the transcription mechanism of *Mononegavirales* in which individual ORFs are sequentially transcribed [65]. The viral polymerase recognizes the 3′ end of the genome and transcribes a short leader RNA followed by the viral genes. Since the polymerase complex is not always successful at re-initiate transcription of the subsequent gene this results in a 3′–5′ transcription gradient [65].

AealbAV does not share detectable sequence identity at nucleotide level with Aedes anphevirus identified in *Ae. aegypti* and it is more closely related to a virus recently discovered in *C. pipiens* mosquitoes [42]. We did not find evidence of AealbAV infecting *Ae. aegypti* mosquitoes, suggesting that this virus might be highly host-specific. The putative anphevirus-like NIRVs we found in *Ae. albopictus* genomes and those found in the *Ae. aegypti* [46] suggest that species of this viral group share a long evolutionary history with *Aedes* mosquitoes.

AealbAV infection seems relatively common as it is found in *Ae. albopictus* laboratory colonies and wild populations from different geographical locations collected over several years. The laboratory colonies from the USA and Italy were established from field collected eggs or larvae and pupae. It is likely that the source samples of these colonies were infected with AealbAV and that the virus has been maintained across generations likely through vertical transmission as shown for other ISVs [66,67,68]. AealbAV is present in *Ae. albopictus* samples from different life stages including eggs, larvae and adults, and in libraries from different tissues including heads, thorax, antennae, palpi, Malpighian tubules and ovaries, suggesting that AealbAV has a broad tissue tropism. We did not find AealbAV in RNA-Seq data generated from *Ae. albopictus* cell lines. The high coverage of AealbAV in several samples from embryos/eggs further supports the vertical transmission of this virus. The possible physiological effects of AealbAV on *Ae. albopictus* tissues warrant further investigation. The fact that AealbAV was found in laboratory colonies and at a relatively high abundance suggests that it is unlikely that AealbAV has a profound negative impact on the host fitness or that it is associated with severe disease in *Ae. albopictus*. Nevertheless, it would be important to determine the impact of AealbAV and of other ISVs on *Ae. albopictus* fitness especially in the context of population-based control strategies (e.g., SIT) where the fitness of released individuals is crucial for the success of the approach.

We have shown AealbAV is targeted by the *Ae. albopictus* RNAi response with the production of both AealbAV-specific vsiRNAs and vpiRNAs. This indicates that AealbAV is efficiently targeted by *Ae. albopictus* Dicer−2 protein and by the ping-pong amplification loop, and suggests that AealbAV was actively replicating in these mosquitoes. Interestingly, some samples in which we observed AealbAV-derived small RNAs were experimentally infected with CHIKV. The fact that replicating AealbAV was found in these samples suggests that the two viruses can co-infect *Ae. albopictus*. It would be important to determine whether AealbAV has some modulating effects on CHIKV or other arboviruses, and if co-infection with AealbAV can influence *Ae. albopictus* vector competence. AealbAV miRNAs were detected also in laboratory colony mosquitoes experimentally infected with three ISVs. In future it would be important to assess AealbAV presence/absence in strains and populations used to conduct experiments aimed at determining viral infection parameters.

The identification and characterization of AealbAV in *Ae. Albopictus* will now allow the recognition of this ISV infection in mosquito populations and laboratory strains. The discovery of AealbAV in *Ae. albopictus* opens up opportunities to determine its potential physiological effects on this medically important mosquito species, and to assess its potential utility as a transmission reducing agent.

## Figures and Tables

**Table 1:** Details of the *Ae. albopictus* RNA-Seq and miRNA-Seq libraries in which AealbAV was identified.

SRA Study	Number of Libraries	Origin	Lab Colony Location	Sampling Year for Colony Establishment	Collection Site	Sampling Time/(Lab Colony Generation)	Library Type	Library Layout	Evidence	Max Depth of Coverage (% Breadth of Coverage)	Tissue	Sex
SRP012105	12	Lab colony	Georgetown University (USA)	2008	Manassas (USA)	-/(F10)	RNA-Seq	Paired (Illumina)	Assembled genomes	361.5 (100)	Embryos	Mixed
SRP018112	17	Lab colony	Georgetown University (USA)	2008	Manassas (USA)	-/(F13)	RNA-Seq	Paired (Illumina)	Assembled genomes	57.4 (100)	1st instar pharate larvae (whole eggs)	Mixed
SRP050258	16	Lab colony	Georgetown University (USA)	2013	Manassas (USA)	2013/(F3)	RNA-Seq	Paired (Illumina)	Assembled genomes	27.7 (100)	Adult whole body	Female
SRP096579	8	Lab colony	Georgetown University (USA)	2010	Manassas (USA)	-/(F12)	miRNA-Seq	Single (Illumina)	Mapped reads	2.6 (73)	Whole Eggs (Pharate Larvae)	Mixed
SRP007714	2	Lab colony	Georgetown University (USA)	2008	Manassas (USA)	2010/(F5)	RNA-Seq	Single (454)	Mapped reads	4.0 (64)	Oocytes	Female
SRP228299 *	2	Lab colony	Institut Pasteur (FRA)	2008	Kawasaki (JPN)	2015/-	miRNA-Seq	Single (Illumina)	Mapped reads	49.8 (99)	Whole body, ovaries	Female
SRP071220	8	Lab colony	Sapienza University (ITA)	2012	Rome (ITA)	2013/-	RNA-Seq	Paired (Illumina)	Assembled genomes	169.3 (100)	Whole body, heads, antennae, maxillary palps	Male/Female
SRP056407	20	Lab colony (MRA-804)	The Ohio State University (USA)	na	Florida (USA)	2012/-	RNA-Seq	Paired (Illumina)	Assembled genomes	33.6 (100)	Malpighian tubules	Female
SRP034701	18	Lab colony (MRA-804)	The Ohio State University (USA)	na	Florida (USA)	2012/-	RNA-Seq	Single (Illumina)	Mapped reads	3.7 (85)	Malpighian tubules	Female
SRP008316 **	5	Lab colony	Fralin Life Science Institute (USA)	na	na	2012/-	miRNA-Seq	Single (Illumina)	Assembled genomes	1228.7 (100)	Whole body, head and thorax	Female
SRP188743	9	Wild-caught	-	-	Guangzhou (CHN)	2017	miRNA-Seq	Single (Illumina)	Mapped reads	4.8 (77)	Adult Whole body, larvae	Mixed
PRJNA602498	1	Wild-caught	-	-	Arco (ITA)	2011	RNA-Seq	Paired (Illumina)	Assembled genomes	129.4261 (100)	Antennae	Mixed
PRJNA602498	1	Wild-caught	-	-	Trento (ITA)	2011	RNA-Seq	Paired (Illumina)	Assembled genomes	512.9825 (100)	Antennae	Mixed
PRJNA602498	1	Wild-caught	-	-	Ban Rai (THA)	2012	RNA-Seq	Paired (Illumina)	Assembled genomes	27.8252 (100)	Antennae	Mixed
SRP266553	14	Wild-caught	-	-	Ticino (CHE)	2019	DNA-Seq	Single (Illumina)	Assembled genomes	21.5 (100)	Adult whole body	Mixed

AealbAV was identified in several samples from laboratory colonies and wild-caught mosquitoes worldwide. “Number of libraries” corresponds to the number of libraries harboring AealbAV in the corresponding SRA study (See Supplementary Table S1 for details of each library); “Origin” indicates the provenance of the mosquito samples, either a laboratory colony or wild-caught. “Lab colony location” corresponds to the location of the mosquito colony (only for laboratory colonies); “Sampling year for colony establishment” indicates the year in which the source material for establishing the colony was collected, if this information was available (only for laboratory colonies); “Collection Site” corresponds to the sampling site for wild-caught samples. For laboratory colonies, it corresponds to the collection site of the wild source samples used for establishing the colony. “Sampling time/(lab colony generation)” refers to the sampling time for generating the sequencing libraries. The number of generations of the laboratory colonies is reported in brackets if known. “Library type” and “Library layout” indicate the sequencing library type and layout. “Evidence” indicates whether AealbAV reads from AealbAV positive libraries were de novo assembled into contigs or only mapped to the AealbAV representative genome. “Max depth of coverage (% breadth of coverage)” indicates the max depth of coverage found among the individual libraries and the corresponding breadth of coverage, which is reported in brackets; “Tissue” and “Sex” indicate the body tissues/life stage and the sex of the mosquito samples. Multiple tissues are listed if distinct libraries within the same study derived from different tissues (i.e., they are not pools of different tissues). “na” indicates that the information was unavailable. * samples experimentally infected with 3 insect-specific viruses. ** samples experimentally infected with chikungunya virus (CHIKV).

**Table 2:** Nucleotide and amino acid identities of AealbAV predicted open reading frames (ORFs) with corresponding genes of the most closely related viruses.

	Serbia Mononega-Like Virus 1	Culex Tritaeniorhynchus Anphevirus	Culex Mononega-Like Virus 2	Guadeloupe Mosquito Mononega-Like Virus	Aedes Anphevirus
ORF1 (N)	67.8 (43)/**55.3 (98)**	-/**29.9 (50)**	-/-	-/-	-/-
ORF2 (STM)	-/-	-/-	-/-	-/-	-/-
ORF3 (G1)	-/**39.6 (78)**	-/-	-/-	-/-	-/-
ORF4 (G2)	70.4 (91)/**74.2 (99)**	74.9 (12)/**41.9 (93)**	65.9 (16)/**43.7 (96)**	65.6 (11)/**43.7 (96)**	-/**42.0 (96)**
ORF5 (ZnF)	-/-	-/-	-/-	-/-	-/-
ORF6	-/-	-/-	-/-	-/-	-/-
ORF7 (RdRp)	71.1 (70)/**69.0 (99)**	64.9 (13)/**39.2 (95)**	64.7 (12)/**41.7 (96)**	-/**41.6 (97)**	-/**36.5 (96)**

Bold values: amino acid identity; non bolded values: nucleotide identity. Values in parentheses correspond to the percentage of query coverage. “-” indicates no detected similarity. Values of alignments below 10% of query coverage are not reported. Serbia mononega-like virus 1, accession number MT822181.1; Culex tritaeniorhynchus Anphevirus, accession number LC514054.1; Culex mononega-like virus 2, accession number MF176332.1; Guadeloupe mosquito mononega-like virus, accession number MN053736.1; Aedes anphevirus, accession number MH037149.1.

## Supplementary Materials

The following are available online at https://www.mdpi.com/1999-4915/12/11/1264/s1, Figure S1: Distribution and mapping profiles of AealbAV-specific miRNAs in small RNA libraries of *Ae. albopictus* samples infected with CHIKV, Table S1: Mapping statistics and details of the sequencing libraries positive for AealbAV, Table S2: Putative anphevirus-like NIRVs in *Ae. Albopictus* assemblies.
