# Supplementary material for: A Novel Anphevirus in Aedes albopictus Mosquitoes Is Distributed Worldwide and Interacts with the Host RNA Interference Pathway
Source: Viruses. 2020 Nov 6;12(11):1264. doi: 10.3390/v12111264 (PMC7694661; doi:10.3390/v12111264)
Supplement: Supplementary file 1 [file viruses-12-01264-s001.zip › Supplementary_files/Supplementary_Note.pdf]

Supplementary Note

Description of AealbAV ORFs

ORF1 (N protein) of AealbAV is predicted to encode a 448-aa protein and has its closest match with the nucleoprotein of the recently published Serbia mononega-like virus 1 (amino acid identity: 55.30%, query cover 98%). It shares approximately 30% pairwise amino acid identity (~50% query coverage) with the nucleoproteins of other anphevirus-like species (Table 2). ORF2 encodes a 123-aa protein with no detectable similarity to known proteins within the NCBI non-redundant database. No matches were found with HHPred and HMMER as well. Two transmembrane domains were predicted with TMHMM suggesting that ORF2 encodes a highly divergent protein corresponding to the STM protein found in other anpheviruses.

ORF3 and ORF4 encode two putative 533- and 638-aa glycoproteins respectively. ORF3 has amino acid similarity with the corresponding protein of Serbia mononega-like virus 1 (GenBankID: QNS17448.1, identity: 39.6%, query cover: 37%). ORF4 has its closest match with the glycoprotein of Serbia mononega-like virus 1 as well (GenBankID: QNS17449.1, identity: 74%, query cover: 99%) and shares around 43% sequence identity with glycoproteins of various mononegaviruses including Culex mononega-like virus 2, Culex tritaeniorhynchus Anphevirus, Guadeloupe mosquito mononega-like virus and Aedes anphevirus (Table 2). TMHMM predicts two transmembrane domains towards the C-terminus of ORF4 predicted protein (TM1:556-578; TM2:587-606). There are 4 putative O-linked glycosylated sites on ORF4 (at 287, 446, 449 and 619 aa position) according to NetOGlyc analysis.

Close to the L protein, ORF5 encodes a short predicted protein of 46 aa with a Zinc-like finger (ZnF) domain that was previously observed in other anpheviruses [46, 51]. A small putative ORF encoding a 26-aa protein is located between ORF5 and the L protein. In this genomic region, a putative ORF with a transmembrane domain in the C terminus has been described in Aedes anphevirus (AeAV) (ORF6, 37 aa) [46]. The AealbAV protein is 11 aa shorter and does not seem to contain any transmembrane domain. Nevertheless it is observed across all the de novo assembled AealbAV strains.

The last ORF (ORF7) encodes the RNA-dependent RNA polymerase (RdRP) protein (L), a large and highly conserved protein present in all mononegaviruses, which consists of the RdRP, the mRNA capping and mRNA guanine-7-methyltransferase domains. The AealbAV 2054-aa L protein shares 69% identity with the RdRP of Serbia mononega-like virus 1 identified in *Culex pipiens* mosquitoes, and between 36-42% identities with the RdRPs of anphevirus-like species identified in various mosquitoes, including Culex tritaeniorhynchus Anphevirus (LC514054.1), Aedes anphevirus (MH430651.1), Culex mononega-like virus 2 (MF176332.1) and Guadeloupe mosquito mononega-like virus (MN053736.1) (Table 2).
