# Supplementary figures and images for: A Novel Anphevirus in Aedes albopictus Mosquitoes Is Distributed Worldwide and Interacts with the Host RNA Interference Pathway
Source: Viruses. 2020 Nov 6;12(11):1264. doi: 10.3390/v12111264 (PMC7694661; doi:10.3390/v12111264)

SRR346385

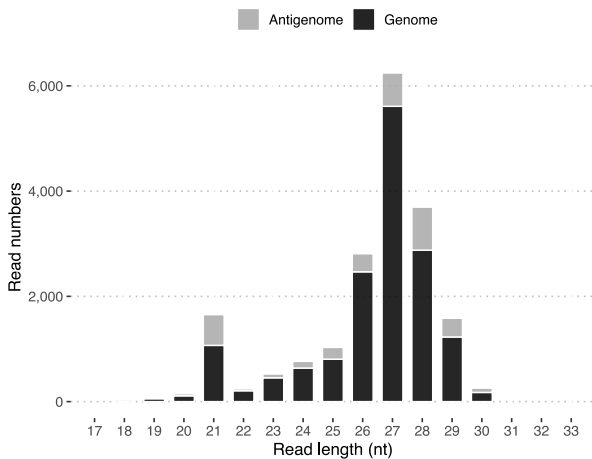

SRR346385 - 21 nt

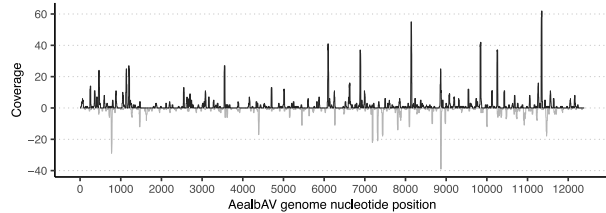

SRR346385 - 27 nt

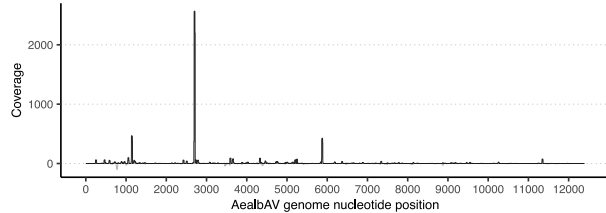

SRR346386

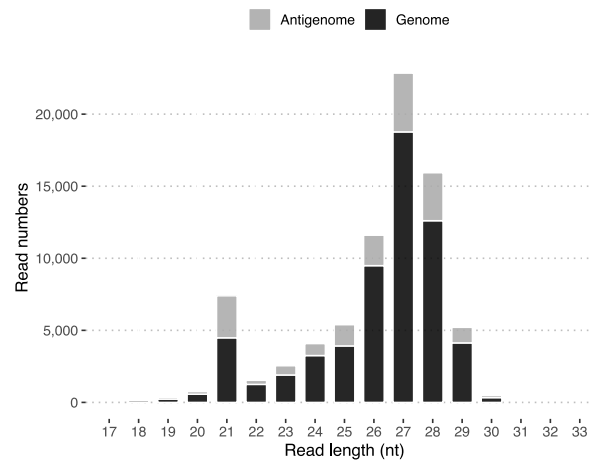

SRR346386 - 21 nt

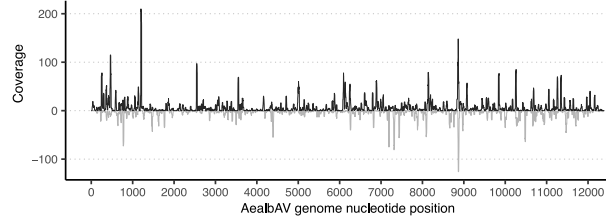

SRR346386 - 27 nt

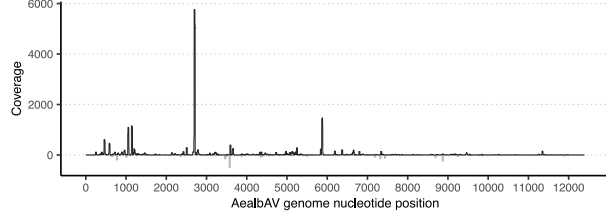

SRR346388

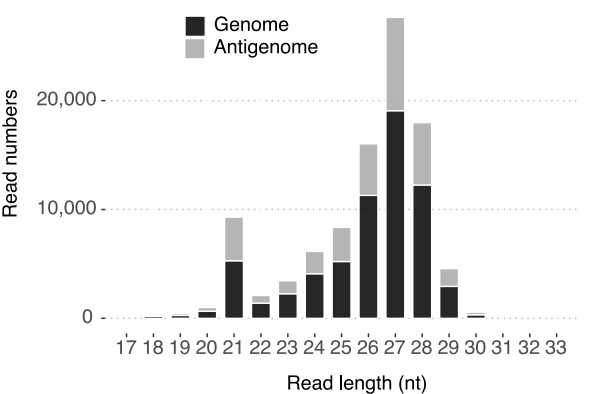

SRR346388 - 21 nt

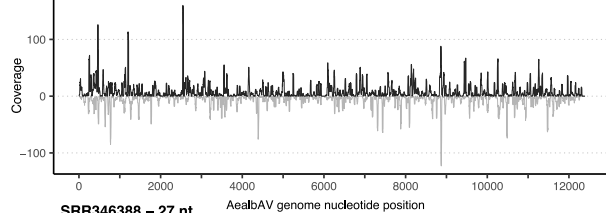

SRR346388 - 27 nt

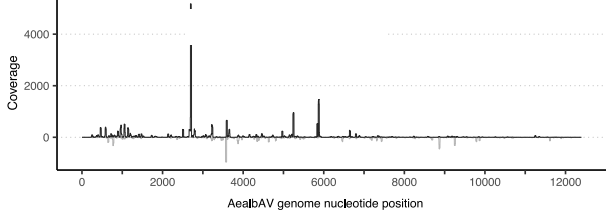

Supplement: Supplementary file 1 [file viruses-12-01264-s001.zip › Supplementary_files/Suppl_Figure_1.pdf]
